# Contrast-Induced Nephropathy in Interventional Cardiology: Incidence, Risk Factors, and Identification of High-Risk Patients

**DOI:** 10.7759/cureus.51283

**Published:** 2023-12-29

**Authors:** Naltin Shuka, Endri Hasimi, Artan Kristo, Leonard Simoni, Taulant Gishto, Ervina Shirka, Elizana Zaimi (Petrela), Artan Goda

**Affiliations:** 1 Cardiovascular Medicine, University Hospital Center "Mother Teresa", Tirana, ALB; 2 Cardiovascular Diseases, University Hospital Center "Mother Teresa", Tirana, ALB; 3 Public Health, Faculty of Medicine, University of Medicine, Tirana, Tirana, ALB

**Keywords:** risk factors, n-acetyl cysteine, hydration, preexisting renal lesion, contrast-induced nephropathy (cin)

## Abstract

Aim: This study aimed to study contrast-induced nephropathy (CIN) or more recent nomenclature contrast-associated acute kidney injury (CI-AKI) in patients undergoing percutaneous coronary procedures, evaluating CIN incidence, risk factors (RFs), and high-risk patients with CIN.

Methods: This is a prospective, observational, unicentric trial of patients who underwent coronary angiography and/or percutaneous coronary intervention (PCI) in the University Hospital Center (UHC) “Mother Teresa” in Tirana, Albania, during 2016-2018. CIN was defined as an increase of 25% and/or by 0.5 mg/dL of serum creatinine (SCr) and high-risk patients with CIN as an increase by 50% and/or by 2 mg/dL and/or need for dialysis compared to the basal pre-procedural values. We evaluated RFs for CIN: preexisting renal lesion (PRL), heart failure (HF), age, diabetes mellitus (DM), anemia, and contrast quantity.

Results: The incidence of CIN resulted in 14.4%. HF, PRL, and age ≥65 years resulted in independent RFs for CIN, whereas anemia, DM, and contrast quantity >100 mL did not. PRL proved to be the most important RF for CIN, whereas HF was the only independent RF for high-risk CIN patients.

Conclusions: The incidence of CIN coincides with the results in the literature. PRL, HF, and age ≥65 years resulted in independent RFs for CIN; more and larger trials are needed to evaluate DM, anemia, and contrast quantity related to their impact on CIN. High-risk patients with CIN represent the most problematic patients of this pathology.

## Introduction

Contrast-induced nephropathy (CIN) is a possible complication related to contrast medium administration in percutaneous coronary interventions (PCIs). It is reported that CIN represents the third most common cause of acute renal failure (ARF) (after acute renal hypoperfusion and drug nephrotoxicity) and is responsible for 11% of these ARF cases [1]. CIN represents an acute exacerbation of the renal function in 48-72 hours after a procedure using a contrast medium, widely defined as an increase in serum creatinine (SCr) by 0.5 mg/dL or 25% compared to the basal pre-procedural values. CIN is a transient process in most cases, and the renal function returns to normal up to seven to 14 days after contrast administration.

Risk factors (RFs) for CIN are still under identification and not yet well clarified. The most important ones include pre-existent renal lesions, diabetes mellitus, advanced age, congestive heart failure, contrast type and volume, concomitant use of other nephrotoxic agents, and dehydration [1]. Other RFs that are being studied are female gender, peripheral vascular disease, and hypertension [2]. It has been observed that patients developing CIN have increased mortality [3], more late cardiovascular events after PCI, an increased incidence of myocardial infarction (MI), and target vessel revascularization in one year, longer hospitalization, and financial cost. Identifying high-risk CIN patients (serious renal disease (SRD)) represents a true challenge because this group of patients is associated with important post-procedural complications and an increase of mortality by three- to five-folds [3,4]. The criteria of one of the most used scores, i.e., the Brown score regarding high-risk CIN, is an increase of SCr 48 hours after the contrast medium procedure by (a) 50%, (b) by 2 mg/dL compared to the basal value, and (c) the need for first-time dialysis [5].

Although CIN requiring dialysis is relatively rare, its impact on patients’ prognosis is considerable, with a high in-hospital and one-year mortality [3,4]. In contemporary studies, CIN requiring dialysis develops maximally in 4% of patients with an already compromised renal function and 3% of patients undergoing PCI for acute coronary syndrome [6].

CIN incidence varies from 3.1 to 31%. Such a high variability depends on several factors, but it indirectly shows inclarity in the final definition of this pathology. Studies on CIN differ from the marker used for the renal function (e.g., SCr, cystatine, and lipocalins), biochemical indicators (serum creatinine and glomerular filtration rate), the day of measuring the initial pre-procedural creatinine and the one after the procedure (24-48/72 hours or up to five to seven days), and the absolute or relative increase in creatinine to define CIN. Some studies even questioned the existence of such a pathology, which suggests that the cause of the decrease in the renal function might not be the administration of the medium contrast [7].

Critics of the existence of CIN have created the term “renalism,” which refers to the avoidance of contrast in patients with renal dysfunction, causing a failure to perform important studies and leading to harm; in the current era of improved contrast dyes, renalism poses a greater risk to patients than does “contrast nephropathy” [8].

Based on the guideline by Kidney Disease Improving Global Outcomes, Acute Kidney Injury (KDIGO AKI, 2012) [9], an individual who develops changes in the renal function after intravascular administration of the contrast medium should be evaluated for this acute renal damage and for every other cause of such damage, whereas the European Renal Best Practice (ERBP) Work Group strongly recommends that individuals showing an increase in SCr in accordance with CIN after intravascular contrast medium administration should be evaluated for other possible causes that might have led to renal function alteration [10].

It is exactly in the frame of this inconclusive thoughts about CIN and its impact on mortality and morbidity that we did our study.

## Materials and methods

We enrolled in this trial patients who underwent coronary interventional procedures in the Cath Lab in University Hospital Center (UHC) “Mother Teresa” from January 2016 to December 2018. The study complies with the Declaration of Helsinki and was approved by the Local Ethics Committee of the hospital. Informed consent was taken from every patient who underwent coronary angiography. The patients were divided into three groups: Group I, the main group who had creatinine measured pre-procedure and 48-72 hours post-procedure; Group II, who had creatinine values measured pre-procedure and 24 hours post-procedure; and Group III, who had creatinine values measured pre-procedure and 24 and 48/72 hours post-procedure.

All patients who had their creatinine measured before the invasive procedure and 48 or 72 hours after the procedure (some of them even 24 hours after the procedure) were included. Meanwhile, all patients under hemodialysis and patients whose creatinine could not be measured at least 24 or 48/72 hours were excluded.

The primary endpoints were considered for the evaluation of the incidence of CIN in 48/72 hours after the invasive coronary procedure: the RFs for CIN (preexisting renal lesion (PRL), age, diabetes mellitus (DM), heart failure (HF), contrast volume, and anemia), the high-risk CIN patients (CIN with an important deterioration of the renal function), the patients who required dialysis for the first time, and the absolute increase in creatinine value by ≥2 mg/dl or increase by ≥50% compared to the pre-procedural level.

The secondary endpoints were considered for the evaluation of the incidence of CIN in 24 hours after the procedure and its predictive value: the role of glomerular filtration rate (GFR) in evaluating the incidence of CIN and the role of pre-procedural SCr as an RF for (SCr >1.5 mg/dL).

The increase in creatinine in 48 or 72 hours after the invasive coronary procedure by a relative value of ≥25% or by an absolute value of ≥0.5 mg/dL compared to the last pre-procedural value was considered CIN. We analyzed the incidence of CIN even in 24 hours with the same criteria. Most of the patients received Ultravist 370 (iopromiode), a non-ionic low-osmolality contrast medium. We monitored the RFs for CIN (age >65, DM, PRL, anemia, systolic HF, and contrast volume used), and we evaluated the significance of each of them.

We used the Cockcroft-Gault formula to calculate the estimated glomerular filtration (eGFR). Pre-existing renal lesion (PRL) was defined as an eGFR <60 mL/min and SCr >1.5 mg/dL. Anemia was considered hemoglobin (Hb) <12 mg/dl pre-procedural. HF patients included only those with systolic HF, an EF <50% pre-procedure. Cut-off age was ≥65 years. Diabetic patients were considered those already known or discovered to have diabetes during the hospitalization.

Statistical analysis

The entire statistical analysis was developed using IBM SPSS Statistics for Windows, version 20 (released 2011; IBM Corp., Armonk, New York, United States). For all categorical variables (nominals including binary/dicotomic and ordinal), we calculated absolute numbers and their respective percentages. For all numerical variables, when data undergo a normal distribution, we calculated the average value ± standard deviations. The chi-square test and Student's t-test calculated the differences between groups regarding variables. To evaluate the incidental connection between the dependent and independent variables, we used the binary logistic regression analysis. We calculated odds ratio (OR) and confidence interval of 95%. The receiver operating characteristic (ROC) curve was utilized in evaluating diagnostic tests (sensitivity and specificity). The data were presented through simple and complicated tables and through various charts (e.g., bar diagram/box-plot/pie charts). The value p ≤ 0.05 was considered significant.

## Results

**Figure 1:**
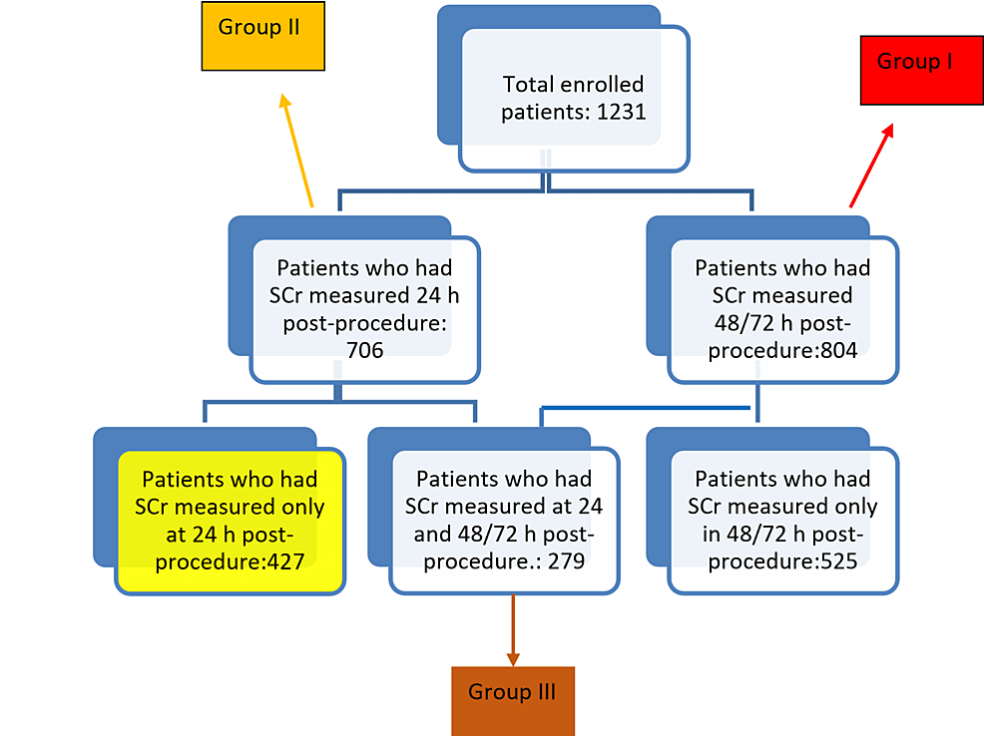
Flowchart of the patients included in the study

**Figure 2:**
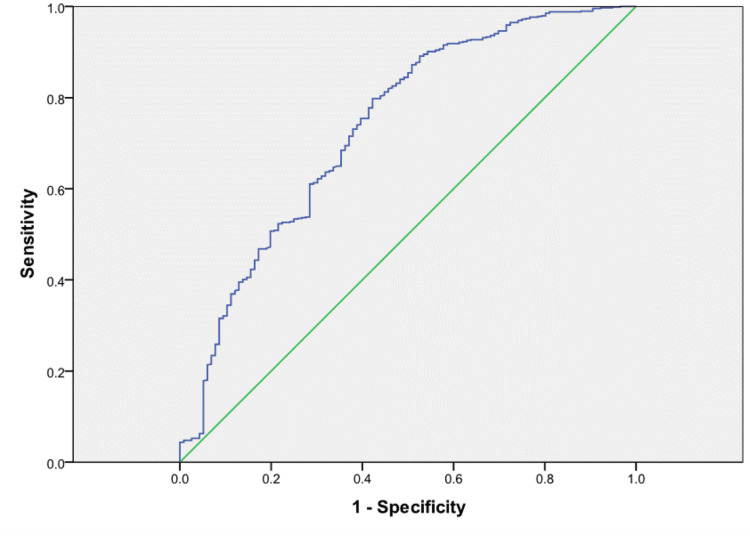
ROC curve of the internal validity of our predictive value The eGFR before the procedure (less than 60 ml/h) as a predictor of CIN in 48 hours showed an area under the curve of 74%, which was considered as a very good value (significant). Diagonal segments are produced by ties.

We enrolled 1,231 patients in total, divided into three study groups: Group I, the main study group with 804 patients; Group II, with 706 patients; and Group III, with 279 patients (Figure 1). The incidence of CIN in 48 or 72 hours based on GFR reduction criteria by ≥25% in 48/72 hours compared to the basal value resulted in 9.07%, lower than the incidence of CIN based on its classic definition. The positive predictive value (PPV) and negative predictive value (NPV) were 100% and 94.12%, respectively, with a low sensitivity of 62.93% but a high specificity of 100% (p < 0.001). The internal validity of our predictive model for eGFR as an RF for CIN is also represented as ROC curves, where we can see that it is significant (Figure 2).

Comparing the basal patients’ characteristics with those who developed CIN (116 patients) and those who did not develop CIN (688 patients), there is a significant difference in favor of age ≥65 years old in the group who developed CIN (53 patients (45.70%) versus 245 patients (35.60%); p = 0.025) (Table 1).

**Table 1:** Basal patient characteristics of patients developing and not developing CIN CIN: contrast-induced nephropathy, PCI: percutaneous coronary intervention, CABG: coronary artery bypass graft surgery, COPD: chronic obstructive pulmonary disease, HTN: hypertension, PAD: peripheral artery disease, BMI: body mass index

Variables		CIN in 48		Total (N = 804)	p
		Yes (N = 116)	No (N = 688)		
		No. (%)	No. (%)		
Gender	M	88 (75.90%)	512 (74.40%)	600 (74.60%)	0.42
	F	28 (24.10%)	176 (25.60%)	204 (25.40%)	
Smoker	No	85 (73.30%)	462 (67.20%)	547 (68.00%)	0.114
	Yes	31 (26.70%)	226 (32.80%)	257 (32.00%)	
Previous PCI	No	104 (89.70%	627 (91.10%)	731 (90.90%)	0.357
	Yes	12 (10.30%)	61 (8.90%)	73 (9.10%)	
Previous CABG	No	112 (96.60%)	665 (96.70%)	777 (96.60%)	0.563
	Yes	4 (3.40%)	23 (3.30%)	27 (3.40%)	
COPD	No	113 (97.40%)	671 (97.50%)	784 (97.50%)	0.569
	Yes	3 (2.60%)	17 (2.50%)	20 (2.50%)	
Age ≥65 years	No	63 (54.30%)	443 (64.40%)	506 (62.90%)	0.025
	Yes	53 (45.70%)	245 (35.60%)	298 (37.10%)	
HTN	No	19 (16.40%)	155 (22.60%)	174 (21.70%)	0.082
	Yes	97 (83.60%)	532 (77.40%)	629 (78.30%)	
PAD	No	115 (99.10%)	679 (98.70%)	794 (98.80%)	0.565
	Yes	1 (0.90%)	9 (1.30%)	10 (1.20%)	
BMI		26.54±3.43	26.79±3.60	26.64±3.51	0.48

Incidence of CIN

The incidence of CIN in 48 or 72 hours, based on the classic definition, resulted in 14.4% (Group I); in the subgroup of patients without RFs, it was 9.1%, whereas in the subgroup of patients with at least one RF, it resulted in 15.3%, which was significant (p = 0.05) (Table 2).

**Table 2:** Incidence of contrast-induced nephropathy CIN: contrast-induced nephropathy, RF: risk factor

Variable	All- CIN	No RF (N = 120)	RF (N = 684)	P value
CIN incidence % (number)	14.4% (116 )	9.1% (11)	15.3% (105)	0.05

The incidence of CIN in 48 or 72 hours based on GFR reduction criteria by ≥25% in 48/72 hours compared to the basal value resulted in 9.07%, lower than the incidence of CIN based on its classic definition. PPV and NPV were 100% and 94.12%, respectively, with a low sensitivity of 62.93% but a high specificity of 100% (p < 0.001). The internal validity of our predictive model for eGFR as an RF for CIN is also represented as ROC curves, where we can see that it is significant (Figure 2).

**Figure 3:**
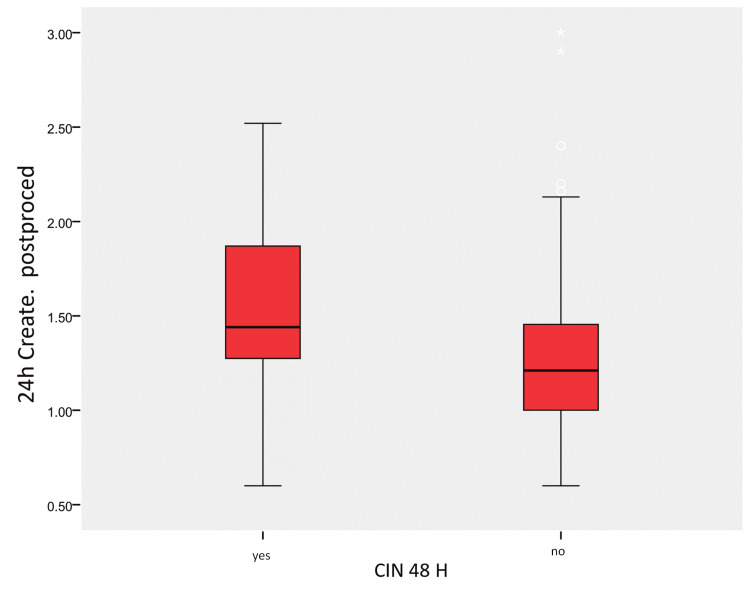
Relation between creatinine in 24 hours and CIN in 48 hours The mean values are reported, while the central line indicates the 50th percentile (median value). The box contains values between the 25th and 75th percentiles, and the whiskers denote the values between the 5th and 95th percentiles.

In Group III, CIN in 24 hours resulted to have a sensitivity and specificity for CIN 48/72 hours of 64.7% and 91.7%, respectively, with a PPV of 63.5% and NPV of 92.1% (Table 3 and Figure 3).

**Table 3:** CIN analysis in Group III CIN: contrast-induced nephropathy

Variables		CIN-48 h (N = 279)		Total (N = 279)	p
		Yes (N = 51, 18.2%)	No (N = 228, 81.8%)		
CIN-24 h	No	18 (35.30%)	209 (91.70%)	227 (81.40%)	<0.001
	Yes	33 (64.70%)	19 (8.30%)	52 (18.60%)	

RFs for CIN (PRL, age, DM, HF, contrast volume, and anemia)

The analysis showed that except for the subgroup of patients with DM, the incidence of CIN in the other subgroups with the classic RF for CIN is higher than the general incidence of 14.4%: DM = 13.1%, anemia = 18.8%, HF = 15%, age ≥65 vjeç = 17.7%, LRP (GFR <60 ml/min) = 18.9% (Table 4) According to the binary logistic regression, the following resulted in an independent RF for CIN: PRL (GFR <60 ml/min or SCr >1.5 mg/dL before the procedure), age ≥65, and HF (Table 5). Other factors, such as DM, volume contrast, and anemia, did not result as independent RFs for CIN. The most important RF for CIN in our patient population resulted in PRL based on GFR <60 mL/min (OR 1.52, CI 95%: 1.02-2.2, P = 0.039).

**Table 4:** Incidence of CIN in subgroups with the classic risk factors CIN: contrast-induced nephropathy, PRL: preexisting renal lesion, GFR: glomerular filtration rate, HF: heart failure, Hb: hemoglobin

Risk factors for CIN	PRL, based on GFR <60 ml/min	Age ≥65 years	Systolic heart failure (HF)	Anemia (Hb <12 mg/dl)	Diabetes mellitus
No. of patients	195	298	120	159	236
Incidence	18.9%	17.7%	15%	18.8%	13.1%

**Figure 4:**
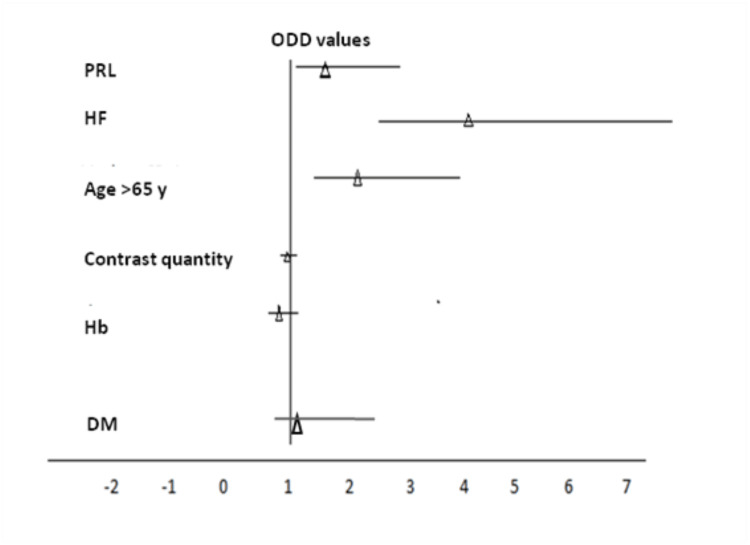
Odds ratio chart of risk factors associated with CIN Forest plot displaying odd ratios (OR) in triangles with 95% Wald confidence limits for CIN (contrast-induced nephropathy) after the univariate analysis (values related to Table 5). The following were included: PRL (preexisting renal lesion), HF (heart failure), age >65, contrast quantity, Hb (hemoglobin), and DM (diabetes mellitus).

Table 5 presents a summary of the association of CIN with RFs and the graphic OR of the RF association with CIN (Figure 4) based on the univariate analysis, whereas Table 6 presents a summary of the multivariate binary logistic regression of the RFs associated with CIN, where PRL based on GFR <60 ml/min resulted as the most important independent RF for CIN (p = 0.039, OR 1.52, CI 95%: 1.02-2.2).

**Table 5:** CIN and risk factors, univariate analysis CIN: contrast-induced nephropathy, DM: diabetes mellitus, PRL: preexisting renal lesion, HF: heart failure, Hb: hemoglobin, OR: odds ratio, CI: confidence interval

Variable	P value	OR	95% C.I.	
DM	0.502	1.16	0.98	2.36
PRL	0.039	1.57	1.02	2.41
HF	<0.001	4.63	2.14	10.05
Age ≥65 years	0.038	1.80	1.37	3.73
Contrast volume	0.753	1.00	0.99	1.01
Hb (mg/dl)	0.171	0.914	0.804	1.040

**Table 6:** Multivariate binary logistic regression: association between CIN and risk factors Variable included in the analysis: HF (heart failure), age ≥65 years, DM (diabetes mellitus), PRL (preexisting renal lesion) based on GFR (glomerular filtration rate)<60 ml/min, PRL based on SCr>1.5 mg/dL, and anemia. CIN: contrast-induced nephropathy, LRP: LDL receptor-related protein, OR: odds ratio

	Variables	OR 95% (CI)	P value*
Step 1^a^	LRP based on GFR	0.749 (0.460-1.220)	0.246
	Age ≥65	1.309 (0.837-2.047)	0.238
	HF	0.958 (0.550-1.670)	0.881
	Diabetes mellitus	0.828 (0.530-1.294)	.407
	Anemia	0.731 (0.457-1.170)	.192
Step 2^a^	LRP based on GFR	0.752 (0.463-1.222)	.250
	Age ≥65	1.307 (0.836-2.043)	.240
	Diabetes mellitus	0.827 (0.529-1.292)	.404
	Anemia	0.732 (0.457-1.171)	.193
Step 3^a^	LRP based on GFR	0.761 (0.469-1.234)	.268
	Age ≥65	1.308 (0.837-2.043)	.238
	Anemia	0.739 (0.462-1.181)	.206
	Constant	5.801	.000
Step 4^a^	LRP based on GFR	1.451 (0.969-2.172)	.071
	Anemia	0.715 (0.449-1.139)	.158
Step 5^a^	LRP based on GFR	1.520 (1.022-2.262)	.039

PRL defined based on SCr >1.5 mg/dL (before the procedure) resulted in an independent RF for CIN. Among the patients with PRL (SCr >1.5 mg/dL), 21.1% (15 out of 71 patients) developed CIN in 48/72 hours, whereas among the patients without PRL, only 13.7% (101 out of 733 patients) developed CIN (p < 0.001). Patients with PRL based on such criteria have a 3.3-fold risk of developing CIN in 48 hours compared to those without CIN. PRL had a low sensitivity and PPV (12.9% and 21.1%, respectively) but a high specificity and NPV (91.1% and 86.2%, respectively).

At the same time, based on the binary logistic regression analysis, the association of DM with a GFR <60 ml/min did not represent an RF for CIN (p = 0.987). When DM and PRL were analyzed together, it was seen that both groups of patients with and without these two factors developed CIN in 14.49% and 14.42% of cases, respectively.

Patients with high-risk CIN

There were no patients who needed dialysis for the first time, but 32 patients (3.9%) had an absolute increase of ≥2 mg/dl or a relative increase of ≥50% of creatinine. This subgroup represents those with high-risk CIN and makes up 27.6% of those who develop CIN. We saw a significant association between those with HF and high-risk CIN, because patients with HF had a 4.5-fold risk of developing high-risk CIN, compared to the patients without HF (OR: 4.49, CI 95%: 2.03-9.95) (Table 7).

**Table 7:** Association of CIN with risk factors and multivariate analysis for patients with high-risk CIN CIN: contrast-induced nephropathy, HF: heart failure, DM: diabetes mellitus, GFR: glomerular filtration rate, PRL, preexisting renal lesion

Variables		Patients with high-risk CIN		OR	CI 95%
		Yes N = 32 (%)	No N = 772 (%)		
Age ≥65	Yes	16 (50.0)	282 (36.5)	1.59	0.71-3.57
	No	16 (50.0)	490 (63.5)	Reference	
HF	Yes	11 (34.4)	80 (10.4)	4.49	2.03-9.95
	No	21 (65.6)	692 (89.6)	Reference	
DM	Yes	8 (25.0)	228 (29.5)	1.37	0.91-5.26
	No	24 (75.0)	544 (70.5)	Reference	
Anemia	Yes	11 (34.4)	148 (19.2)	1.92	0.24-2.16
	No	21 (65.6)	622 (80.8)	Reference	
GFR-based PRL	Yes	10 (31.3)	185 (24.0)	1.19	0.49-2.91
	No	22 (68.8)	587 (76.0)	Reference	
Contrast volume	Yes	18 (56.3)	464 (60.1)	1.35	0.65-2.83
	No	14 (43.8)	308 (39.9)	Reference	

## Discussion

In our research, we observed that 1) the incidence of CIN resulted in 14.%; 2) PRL, age ≥65 years, and HF resulted in independent RFs for CIN, whereas anemia, DM, and volume contrast did not; 3) HF resulted as the most important RF for high-risk CIN; and 4) CIN in 24 hours resulted predictive of CIN in 48/72 hours.

The incidence of CIN resulted similar to other studies, with an average incidence of 14.5% described in the literature for patients who underwent PCIs [3], being higher in the subgroup of patients with RF, such as DM, HF, age ≥65, and PRL, where incidence can vary up to 25-35%, but even lower in those without RFs, varying 1-2%. Such figures were observed as well in our study where the incidence in the group of patients with RF was higher than in that without RFs (15.3% vs. 9.1%, p = 0.05), whereas in the group with PRL, the incidence was found to be as high as 19%.

In an analysis of 15 prospective and retrospective studies (1976-1996), an incidence of CIN varying 3.1-31% was reported [3]. The impact of various definitions of CIN is important and can be demonstrated by the Oxilan Registry results [11]. Efforts have been made to achieve a consensus in defining CIN. In the 2012 guideline by KDIGO AKI, a new definition and qualification of AKI was recommended, based on a combination of RIFLE (Risk, Injury, and Failure; and Loss; and End-stage kidney disease)/AKIN classifications: an increase of SCr by KrS by ≥0.3 mg/dl in 48 hours or an increase in SCr y ≥1.5 folds (>50%) compared to the basal value happening within seven days of the procedure [12]. 

Our study analysis was based on using SCr to calculate the incidence of CIN. However, we also analyzed the role of GFR in calculating the incidence of CIN, being one of the accepted definitions for CIN: RIFLE criteria or European Society of Urogenital Radiology (ESUR), as well as in various studies. It was seen that compared to the calculated incidence through SCr, the incidence of CIN calculated based on GFR was the lowest one (9.07% vs. 14.4%), with a lower sensitivity but a high specificity of 100%. Thus, the question is whether GFR is a better marker of renal function to evaluate acute renal function and consequently of CIN as well. Studies show that GFR is a better marker of renal function in chronic kidney disease (CKD), but patients who undergo contrast medium procedures do not necessarily suffer from CKD, and that is why SCr is still used in evaluating renal function in CIN, despite the recommendation to estimate GFR, especially when evaluating the risk for CIN [13].

Our study showed that creatinine in 24 hours after the procedure seems to predict the development of CIN in 48/72 hours after the procedure. Its evaluation in 24 hours after a contrast medium procedure is rarely used in the literature and its relevance is not well studied. A lot of patients undergoing invasive coronary procedures are dismissed from the hospital 24 hours after the procedure, so this evaluation (in 24 hours) would help the triage of patients.

Even the randomized study PRavastatin Inflammation CRP Evaluation (PRINCE) [14] showed that in 80% of patients with CIN, serum creatinine starts to increase in the first 24 hours after being exposed to contrast medium and almost all patients who progress to serious renal failure (requiring nephrology consultation or dialysis) have an increase in serum creatinine during the first 24 hours. Another study from Ribichini et al. [15] including 216 patients showed that the percentual change in SCr 12 h after contrast medium administration, compared to its basal pre-procedural value, is the best predictor for CIN (p = 0.001).

The results of our study regarding the significant RFs for CIN after coronary angiography/PCI are concordant with the other studies only for some of the RFs. The variability of CIN definition can be one of the main reasons for the inconclusive results. Some studies take into consideration only one of these two criteria used in our study, some others measure it three to five days after the procedure, while some others measure GFR, which could influence both the incidence of CIN and the significance of the RFs [1].

In this study, we defined PRL as eGFR <60 ml, recommended by the Working Group on CIN (CI-AKI Consensus Working Panel [9]. We also studied as a secondary endpoint SCr >1.5 as criteria for PRL, which resulted to be a significant RF for CIN. The multivariate analysis showed that HF was also significant for CIN. These two results are concordant with almost all the other studies. PRL is considered to be the most important RF for CIN in various studies and guidelines. This is related to the fact that patients with PRL have a reduced vasodilator response, which is an important RF for the development of CIN. At the same time, the reduction of GFR in these patients prolongs the elimination of contrast media, thus enhancing its cytotoxic and hemodynamic effects [3,16]. On the other hand, HF is characterized by a decrease in cardiac output, an increase in the vasoconstrictor neurohormonal activity, and continuous renal vasodilation dependent on nitric oxide (NO), which could lead to renal medulla hypoperfusion, increasing the risk for acute renal dysfunction and eventually for CIN. We considered only those with systolic HF, as an RF for CIN, and not those with diastolic dysfunction. Other studies support the idea that diastolic HF is also an independent RF for NIK [3,17].

We note that in our study, the specificity of using increased Scr (>1.5 mg/dL) before the procedure was higher than decreased GRF before the procedure (<60 ml/min) to detect patients developing CIN. An eGFR <60 ml/min before the procedure detects more patients who are going to develop CIN compared with SCR >1.5 mg/dL (32% vs. 12.9%), but an SCr <1.5 mg/dL before the procedure is reassuring at the same level as an eGFR >60/min (NPV of 86.22% and 87.03%, respectively).

PRL based on increased SCr before the procedure is known as a decisive RF for the development of CIN as confirmed also in this study [18]. In a study by Gruberg and Mehran [19], CIN developed in 1/3 of patients who underwent PCI and had a SCr ≥1.8 mg/dL. The Contrast-Induced - Acute Kidney Injury Consensus Working Panel accepted that the risk for CIN becomes clinically important when basal SCr >1.3 mg/dL for men and >1 mg/dL for women, equivalent to eGFR <60 ml/min per 1.73 m^2^ [9].

However, the study showed that the sensitivity of GFR <60 ml/min before the procedure to detect CIN was higher than increased SCr (31.9% vs. 12.9%), whereas in the multivariate analysis, eGFR resulted in the most important RF for CIN. That is why in the definition of PRL as an RF for CIN, it is recommended to estimate GFR before the contrast procedure. The ESC and American guidelines and the KDIGO and ESUR recommend measuring SCr and eGFR before contrast procedures to evaluate the risk for CIN [9].

Age ≥65 years resulted to be significant for CIN. The reasons are multifactorial, including the age-related changes in the renal function (which favors sodium renal retention and loss of water), the presence of aged vessels, the higher probability for other comorbidities that worsen the renal function such as dehydration, CKD especially when treated with angiotensin-converting enzyme inhibitors (ACEIs) or angiotensin II receptor blockers (ARBs), the advanced coronary artery disease, long-term HTN, and DM.

There are limited data to determine the epidemiology of CIN in older patients, whereas accepting older age as an independent RF for CIN is in line with previous studies [1,3].

A meta-analysis [20] of recent studies (22 studies, with 186,455 patients) showed that the total incidence of CIN in the older patients was 13.6% (95% CI 10.1-18.2, I^2 ^= 0.496), whereas in our study, the older patients (>65 years) had a 52% higher risk to develop CIN. The cut-off in our study was 65 years old, which is concordant with the above-mentioned meta-analysis. However, there are other cut-offs used in various studies and scores, such as >70-75 years, whereas in the Mehran risk score for CIN, this value was 75 years.

The fact that we did not find contrast volume >100 ml a significant factor for CIN might be related to the fact that we did not use high volumes of contrast; on average, it is 170±99 ml; in those who developed CIN, 189.22±122.44 ml; and in those who did not develop CIN, 177.37±102.02 ml. The ESC/EACT guideline (2014), to prevent CIN, recommends the following elements as targets for contrast medium: volume <350 ml or <4 ml/kg or a ratio V/CrCL < 3.7:1 (Class I indication).

As regards DM as an independent RF for CIN, which did not result in our study, literature data are inconclusive and contradictory. Although there is a considerable number of studies or panoramic articles from various authors studying CIN, such as Mehran and Nikolsky [1], who recognized DM as an independent and important significant RF for CIN, especially when accompanied by CKD; there are also other studies that showed that DM is not an independent RF for CIN in patients with a normal renal function. DM was found to be an independent RF for CIN when associated with proteinuria, which was not studied in our trial; we did not measure proteinuria [21]. Even ESUR does not consider DM as an RF per se if not associated with renal dysfunction. One hypothesis that can explain such a result might be “preconditioning” of the renal function by diabetes: among the implicated main mechanisms in CIN might be oxidative stress and contrast-induced hypoxia, which are present in diabetic patients through chronic adaptation and might provide some tolerance from acute hypoxic stress and oxidative stress induced by contrast medium, thus modifying the CIN phenotype in diabetic patients [22]. However, you should take into consideration that a diabetic patient with concomitant CKD, even with just proteinuria, represents a high-risk patient to develop CIN [1], reinforcing the idea of carefully evaluating renal function in diabetic patients undergoing invasive procedures with a contrast medium.

Anemia was not found as an independent significant RF for CIN, which is in discordance with other studies. Patients with anemia before the procedure were preliminary treated (even with blood transfusions), thus having acceptable Hb values: in the group who developed CIN, Hb was 12.97±1.7, whereas in those who did not develop CIN, Hb values were 13.18±1.49, which was not significant. This might be one of the reasons why anemia did not result in an independent RF for CIN, in accordance with other studies [23].

Based on our study criteria, 32 patients resulted with high-risk CIN (27.6%, out of the total number of patients who developed CIN), which represents a percentage close to the one reported in the literature for problematic patients after CIN (<1/3 of patients remained with residual renal dysfunction even after seven to 14 days) [24]. Such a percentage has been evaluated according to the protocol only 48/72 hours after the procedure, and we do not have any data regarding patients who have residual renal dysfunction after seven to 14 days to make a more relevant comparison. Compared to the total number of patients studied, the incidence of high-risk CIN resulted in 3.9%, which is higher than that reported in the literature, i.e., 0-1.57% [24], which might be related to the used definition criteria for these patients.

Among the interesting results of our study was the fact that patients with high-risk CIN had systolic HF as the most important RF. However, in this group of patients, only HF resulted as an independent RF. Meanwhile, in the literature, PRL is continuously reported to be the most important RF for high-risk CIN, which coincides with our study results in the multivariate analysis only regarding PRL as an RF for CIN [12,25]. It is accepted that PRL becomes important especially when eGFR reaches <20-40 ml/min, i.e., in moderate and severe kidney diseases. These stages are scored four to six points (Mehran risk score), whereas chronic congestive HF is scored five points, i.e., an important RF for CIN and indirectly for high-risk CIN. Even in the Maioli score [26], HF (EF <45%) and eGFR <44 ml/mn (moderate to severe renal dysfunction) or SCr >1.5 mg/dL are equally scored by two points, giving considerate importance to HF. A lot of studies and articles have shown that HF New York Heart Association (NYHA) functional classification III/IV is associated with a high risk for CIN at around 50% [3,16,27]. We note Rosenstock et al.'s study [28], which concluded that the most important factor for CIN in CKD patients’ population (eGFR <60 ml/min, SCr >1.5 mg/dL) is HF (EF <40%), whereas patients with CKD but without evidence of HF who receive proper hydration have a much lower risk for CIN [28]. Another work that shows the importance of HF in developing high-risk CIN is that of Gruberg et al. [19], which shows that CKD and significant EF reduction were the most important among the studied RFs for high-risk CIN and dialysis.

The same parameters used in this study to determine patients with CIN with significant renal dysfunction have been evaluated by Brown et al. [5]. Other scores (Freeman and Gurm) [29] mainly use dialysis as a severity criteria for high-risk CIN, whereas Bartholomew et al. and Mehran et al. [17,27] used the absolute increase in SCr by 1 mg/dL to define patients with high-risk CIN. These changes together with the differences in the number of patients included in trials may explain the discrepancies in the incidence of high-risk CIN in our study (which is almost twofold higher) compared to that in the literature (3.9% vs. 1.57%).

Study limitations

Some of the patients increased SCr later than 48/72 hours, but we measured SCr after 48/72 hours according to our protocol, based also on our clinical practice. A part of the patients were lost for CIN evaluation. We have not evaluated diastolic HF, which could lead to an incomplete evaluation of the impact of HF on CIN. Proteinuria was not evaluated when evaluating DM as an independent RF for CIN, which would help in making a more complete evaluation of this factor.

## Conclusions

The incidence of CIN in the population of patients undergoing coronary angiography and/or PCI in UHC “Mother Teresa” in Tirana, Albania, was similar to the incidence in most of the other studies, being higher in those with RFs. PRL, HF, and age >65 years are independent RFs for CIN. PRL represents the most important RFs for CIN, in the population of Albanian patients studied. Other studies are required, with a greater number of patients to study the other RFs not confirmed in our study, such as DM, anemia, and contrast volume. Measuring creatinine after 24 hours has a high positive predictive value for the development of CIN in 48/72 hours. Absolute SCr 1.5 mg/dL before the procedure represents a specific predictive indicator for PRL to be an independent RF, but eGFR remains the most sensible marker for CIN. Correcting anemia before a contrast procedure and limiting contrast volume should always be taken into consideration. Patients with high-risk CIN represent a small group of patients, and HF resulted as the main independent RF for this group. CIN is still a debatable entity as regard its definition, importance, and clinical variability. It is important to unify the definition of CIN to better study and manage this pathology. Accepting the KDIGO - AKI criteria would serve this objective at its best.
